# SAHBNET, an Accessible Surface-Based Elastic Network: An Application to Membrane Protein

**DOI:** 10.3390/ijms140611510

**Published:** 2013-05-30

**Authors:** Nicolas Dony, Jean Marc Crowet, Bernard Joris, Robert Brasseur, Laurence Lins

**Affiliations:** 1 Center of Protein Engineering, University of Liège, Institut de chimie B6a, B-4000 Liège, Belgium; E-Mails: n.dony@ulg.ac.be (N.D.); bjoris@ulg.ac.be (B.J.); 2 Numerical Molecular Biophysics Unit, Gembloux Agro-Bio Tech, University of Liège, Passage des déportés, B-5030 Gembloux, Belgium; E-Mails: jmcrowet@ulg.ac.be (J.M.C.); rbrasseur@ulg.ac.be (R.B.)

**Keywords:** molecular dynamics, elastic network, coarse-grained, MARTINI forcefield, membrane, lipids, protein structure, hydrogen bond, accessible surface

## Abstract

Molecular Dynamics is a method of choice for membrane simulations and the rising of coarse-grained forcefields has opened the way to longer simulations with reduced calculations times. Here, we present an elastic network, SAHBNET (Surface Accessibility Hydrogen-Bonds elastic NETwork), that will maintain the structure of soluble or membrane proteins based on the hydrogen bonds present in the atomistic structure and the proximity between buried residues. This network is applied on the coarse-grained beads defined by the MARTINI model, and was designed to be more physics-based than a simple elastic network. The SAHBNET model is evaluated against atomistic simulations, and compared with ELNEDYN models. The SAHBNET is then used to simulate two membrane proteins inserted in complex lipid bilayers. These bilayers are formed by self-assembly and the use of a modified version of the GROMACS tool genbox (which is accessible through the gcgs.gembloux.ulg.ac.be website). The results show that SAHBNET keeps the structure close to the atomistic one and is successfully used for the simulation of membrane proteins.

## 1. Introduction

The study of membrane proteins has become one of the most challenging fields in biology. More than 30% of eucaryotic proteins are membranous and they are the most targeted proteins by common drugs [1]. They are involved in many important processes such as energy production, transport across membrane, or cell-to-cell signaling [2–5], but they only represent less than 1% of the entries in the Protein Data Bank [6,7]. Furthermore, the structure often only partly explains the activity or interaction. Insertion into the membrane and activity of these proteins can also depend on the composition (length of lipids tails, charge of the polar head) and/or asymmetry of the membrane [8–12]. Moreover, membrane proteins can be hard to study *in vitro* due to the difficulty for those proteins to be produced, purified, and reconstituted into membranes [13–17]. Therefore, calculation methods to study membrane protein folding, lipid insertion, or interaction with lipids or proteins are helpful to complement experimental studies and fill in the gaps between the information obtained from the sequence and/or structure, the experimental results and the biological activity [18–23].

Molecular Dynamics (MD) is a method of choice for membrane simulations with lipids, alone or in the presence of proteins [24,25]. MD is currently used to study complex lipid-associated phenomena like membrane protein interaction, vesicle fusion, or curvature-driven lipid sorting. Usually, extended simulations (≥4 μs) are compulsory to observe a wide range of membrane-associated processes. Large systems are also difficult to set up and need a great deal of computational resources. As the time scale of atomistic simulations (AT) is typically in the range of hundreds of nanoseconds, none of these processes can be reached.

Few years ago, the rising of coarse-grained (CG) forcefields has opened the way to longer simulations with reduced calculations times [26–32]. In coarse-grained models, groups of atoms are considered as a single particle. This simplification concomitantly reduces the calculation time while increasing the simulation timescale. In some cases, this approach has proven to be as good as atomistic forcefields in terms of interactions and shows a great ability to simulate complex molecular phenomena associated to lipids [27,28]. The CG MARTINI’s forcefield [27,28] available for GROMACS [33] has been extensively used for lipids and proteins. In this forcefield, one particle represents, on average, four heavy atoms. The different CG particles types interact through Lennard-Jones and Coulomb potentials and bond lengths and angles are maintained by using soft harmonic potentials [27,28,34]. However, with this forcefield, the tertiary structure has to be maintained. Marrink and coworkers have introduced different constraint networks to do so. One of them maintains the conformation by using elastic bonds according to the secondary structures found in the atomistic representation [28]. Another method from Periole *et al.* [35], called ELNEDYN is also based on elastic bonds. Their network definition is as follows: “Two backbone beads are linked by a spring with a force constant K_SPRING_ only if the distance between them in the experimental structure is less than a predefined cut-off, R_c_, and if they are at least separated by two positions in the protein sequence”. They added to that spring network a precise description of each bond and angle of the CG structure according to the atomistic representation. The first method is perfect to maintain the secondary structures but actually fails to maintain the tertiary structure. On the other hand, ELNEDYN can keep the tertiary structure close to the atomistic one, but protein flexibility and thus domain movements could be hindered.

Several groups have tried to improve the structure stability in MARTINI’s forcefield without losing the protein flexibility. For example, Shen *et al.* [36] have used short atomistic simulations to calculate the right constraint’s energy (K_SPRING_) for each string to make a heterogeneous network. More recently, Globisch *et al.* [37] have developed IDEN derived from ELNEDYN. It uses averaged distances calculated from short atomistic simulations instead of crystallographic reference and adds two conditions to the R_c_ cutoff.

Instead of making a global elastic network, or calibrating a heterogeneous network, we have tried to stabilize the structure by using information present in the atomistic structure. We first decided to keep the hydrogen-bond (h-bond) network and then, based on a previous work from the lab on the accessible surface of residues [38], we assumed that if a residue is buried inside the protein, there is a high probability that its interactions with the other residues are important for the global protein stability. Springs between buried residues and their neighbors are therefore added to the h-bond network. These two networks are used in conjunction with the MARTINI’s secondary structure network. Following the description made for ELNEDYN, the hydrogen-bonds elastic network is defined as: “Two backbone beads are linked by a spring with a force constant, K_SPRING_, if their atomistic counter-parts are involved in a hydrogen bond” and the surface accessibility elastic network as: “The Backbone(BB) bead or side-chains (SC) beads from a residue with an accessible surface smaller than a defined cut-off (SA_c_) is linked by a spring with a force constant, K_SPRING_, to all BB beads or SC beads whose distance in the experimental structure is less than a predefined cut-off, R_c_, and if they are at least separated by five positions in the protein sequence”. This definition allows the selection of “buried” residues and to use them to help the MARTINI secondary network to maintain the protein 3D structure by restraining some local interactions inside the protein. The merging of the two networks is called SAHBNET, for Surface Accessibility Hydrogen-Bonds elastic NETwork.

In this study, we show how SAHBNET is calibrated and compared to ELNEDYN in terms of stability on small and larger soluble proteins. The method is also applied to a membrane protein and a larger membrane complex.

## 2. Results and Discussion

### 2.1. Calibration of the Surface Accessibility Hydrogen-Bonds Elastic NETwork (SAHBNET) against Small Soluble Proteins in a Protein/Water System

Using the three soluble proteins studied in the paper presenting ELNEDYN [35], we prepared multiples CG simulations using either the simple MARTINI’s constraints network with and without SAHBNET on backbone beads (BB), or on side-chains beads (SC), or using ELNEDYN; one atomistic simulation for each protein was also computed. We compared their Root Mean Square Deviation (RMSD) and Root Mean Square Fluctuation (RMSF) according to the variation of three variables: SA_c_, R_c_, and K_SPRING_. As already described in many publications [27], the smoothing of the energy landscape in MARTINI forcefields induces a modification of the speed of the particles that can be corrected by applying a factor of four on time. Unless stated otherwise, all times corresponding to CG simulation denoted by a * are real time (4× simulation time).

**Figure 1:**
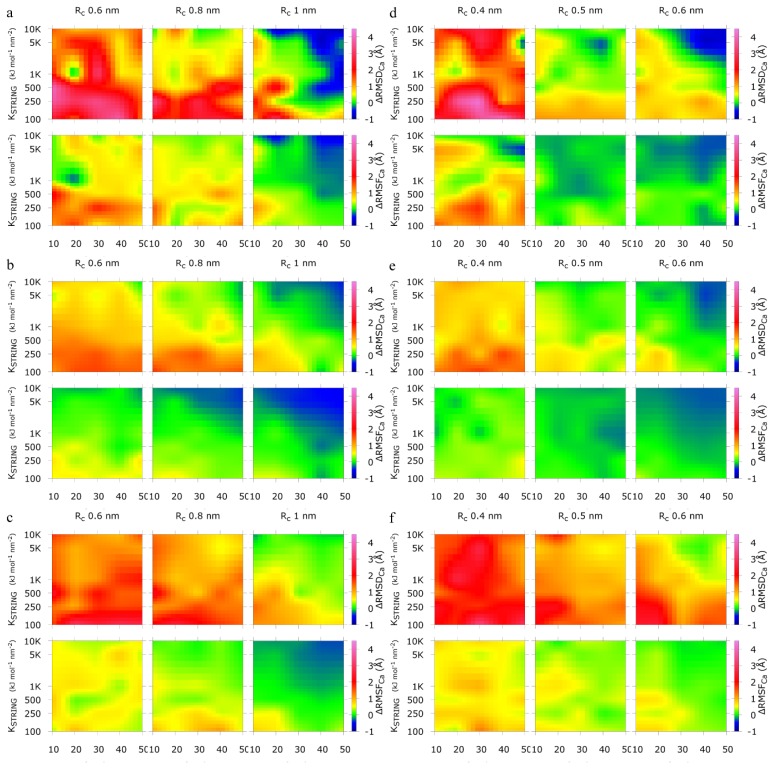
Comparison between atomistic and Surface Accessibility Hydrogen-Bonds elastic NETwork backbone beads (SAHBNET BB)[left column: (**a**–**c**)] and side-chains beads (SC) [right column: (**d**–**f**)] CG simulations of (**a**,**d**) the villin headpiece subdomain, (**b**,**e**) the α-spectrin SH3 domain and (**c**,**f**) the B1 domain of protein G. Value of Delta-Root-Mean-Square Deviation of the Cα (ΔRMSD_Cα_), Delta-Root-Mean-Square Fluctuation of the Cα (ΔRMSF_Cα_) are reported as a function of the restraint energy (K_SPRING_; vertical axis) and the SA cut-off (horizontal axis) for each R_c_ values. Green zones correspond to parameters for the SAHBNET where the CG structure behavior is the closest to the atomistic one. Blue zones, corresponding to negative values, indicate parameters for which the movements of the CG structure are too much hindered by the SAHBNET.

To compare the behavior of the CG structure using SAHBNET to the atomistic one, the Delta-Root- Mean-Square Deviation of the Cα (ΔRMSD_Cα_) and Delta-Root-Mean-Square Fluctuation of the Cα (ΔRMSF_Cα_) have been calculated for the villin headpiece subdomain, the B1 domain of protein G, and the α-spectrin SH3 domain. The results for the three proteins are presented in Figure 1a–c for SAHBNET BB and in Figure 1d–f for SAHBNET SC. In these figures, values of ΔRMSD_Cα_ and ΔRMSF_Cα_ are reported as a function of K_SPRING_ and SA_c_ value for a selected R_c_ cutoff value.

The results show a good overall conservation of the structure of the protein. As expected, the higher the value of the variables, the lower the global deformation of the protein. The blue zones, representing negative values, indicate the value of SA_c_, R_c_, and K_SPRING_ for which the SAHBNET is too stringent and where the CG structure flexibility is compromised compared to the atomistic one. The same results are observed when SAHBNET SC is used. The boundary values that appear to give the best network are: SA_c_ around 30%, R_c_ 0.8–0.9 and 0.4–0.5 nm respectively for SAHBNET BB and SAHBNET SC and K_SPRING_ around 1000 kJ mol^−1^ nm^−2^. The SA_c_ value of 30% is close to the limit usually admitted for buried residue but for some small proteins a higher value is sometime needed to select a “buried” residue. The K_SPRING_ value is in the same range as for ELNEDYN. The R_c_ depends on the SAHBNET type. For BB type, the R_c_ value of 0.9 nm is the same as ELNEDYN. Logically, for SC network, the R_c_ value, around 0.5 nm, is lower as we are linking interacting side-chains.

**Figure 2:**
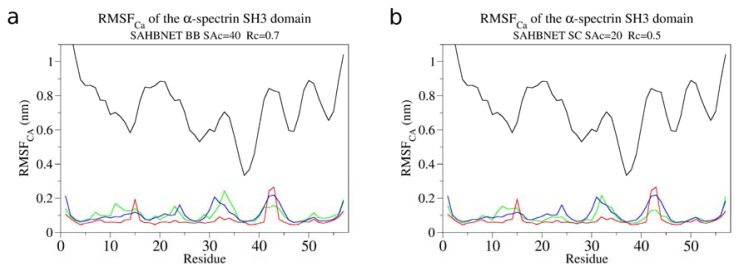
Comparison of the RMSF of the Cα between atomistic (blue), ELNEDYN (red), without network (black) and SAHBNET (green) BB [(**a**) SA_c_ = 40%, R_c_ = 0.7 nm and K_SPRING_1000 kJ/nm^2^] and SC [(**b**) SA_c_ = 20%, R_c_ = 0.5 nm and K_SPRING_1000 kJ/nm^2^] CG for the simulations of the α-spectrin SH_3_ domain.

**Figure 3:**
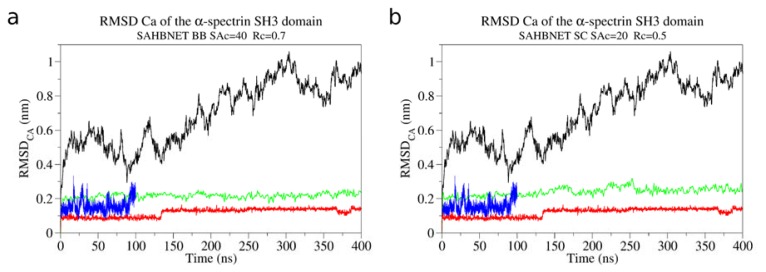
Behavior during longer time scale: The RMSD of the Cα over time is presented for the α-spectrin SH3 with SAHBNET (green) BB [(**a**) SA_c_ = 40%, R_c_ = 0.7 nm and K_SPRING_1000 kJ mol^−1^ nm^−2^] and SC [(**b**) SA_c_ = 20%, R_c_ = 0.5 nm and K_SPRING_1000 kJ mol^−1^ nm^−2^], with ELNEDYN (red), without network (black) or from atomistic simulation (blue).

The RMSF_Cα_ of the α-spectrin SH3 domain per residue using SAHBNET BB and SC are shown on Figure 2a,b respectively. The values are compared to those obtained using ELNEDYN and with an atomistic simulation. Figure 3 clearly shows that a restraint network has to be applied to maintain the structure (compare black curve to the three others). SAHBNET BB or SC fits very well to the atomistic model behavior, especially for the loops. To test if the structure is kept stable over time, we have extended the simulations time up to 400 ns* and compared the RMSD using SAHBNET BB or SC network to ELNEDYN and without additional networks (Figure 3). Without networks, the RMSD shows large variations (over 3 Å) in the protein and no sign of recovery for shorter simulations. With ELNEDYN, the structure is very stable (RMSD around 1 Å) as with the SAHBNET network (2 Å RMSD on average), the atomistic values varying between one and two.

### 2.2. Comparison between the SAHBNET and ELNEDYN Networks

**Figure 4:**
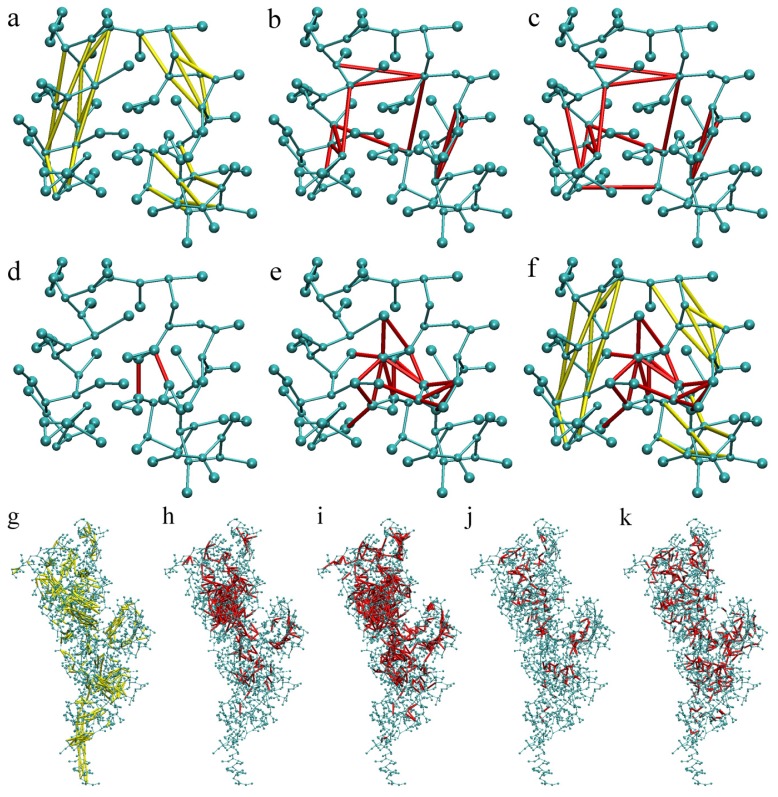
Representation of the SAHBNET network on the villin headpiece subdomain: (**a**) Hydrogen-bond network only; (**b**) BB based network with only SA springs with SA_c_ = 30% and R_c_ = 0.9 nm; (**c**) SA_c_ = 50% and R_c_ = 0.9 nm; (**d**) SC based network with only SA springs with SA_c_ = 30% and R_c_ = 0.4 nm; (**e**) SA_c_ = 30% and R_c_ = 0.5 nm; (**f**) Complete SAHBNET with SA_c_ = 30% and R_c_ = 0.5 nm and the networks on PBP1b; (**g**) Hydrogen-bond network only; (**h**) BB based network with only SA springs with SA_c_ = 5% and R_c_ = 0.9 nm; (**i**) SA_c_ = 30% and R_c_ = 0.9 nm, SA SC based network with (**j**) SA_c_ = 5% and R_c_ = 0.5 nm; (**k**) SA_c_ = 30% and R_c_ = 0.5 nm. Proteins beads are represented in blue, the SA and H-bond springs are represented respectively in yellow and red.

The protein characteristics (number of residues, hydrogen-bonds, and ELNEDYN springs) and the number of springs generated to make SAHBNET depending on the protein, the SA_c_, and the R_c_ values are presented respectively in Tables S1 and S2. As expected, the number of springs increases with the number of residues, SA_c_, and R_c_ values. The number of springs made for ELNEDYN is proportional to the protein size, and overall is much larger than for SAHBNET. ELNEDYN and SAHBNET localization is different (Figure 4). The ELNEDYN network is uniformly present over all the backbone beads, reducing the overall backbone flexibility while SAHBNET stabilizes the protein structure by forcing interactions around the buried residues.

The SA_c_ value depends on the protein. Rationally, a small protein, less prone to have buried residues, may need larger values than larger proteins to induce enough network nucleation spots to stabilize the structure. For larger proteins, like PBP1b, there is already a network at a SA_c_ value of five and using the right SA_c_ and R_c_ values leads to the formation of independent network defining subdomains (Figure 4).

For SAHBNET, the hydrogen-bond network is localized inside alpha-helices and between beta-strands as expected, but can also be found in structured loops. This part of SAHBNET is sometimes redundant with the forcefield secondary structure network in α-helices and with non-bonded interaction defined for beads with hydrogen-bonding capabilities. This part should be further improved in the future.

### 2.3. Testing SAHBNET on a Larger Soluble Protein: The RNA-Dependent RNA Polymerase from Human Rhinovirus 16

**Figure 5:**
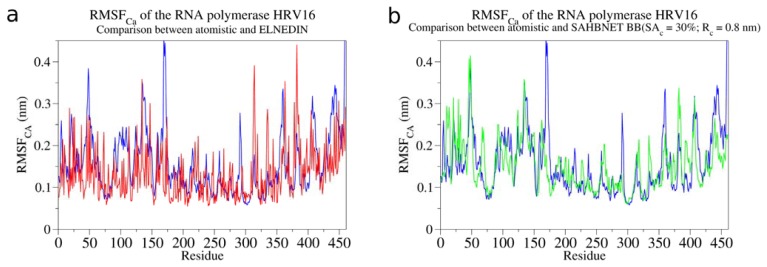
(**a**) Comparison of the RMSF of the Cα between atomistic (blue), and ELNEDYN (red) (**b**) between atomistic (blue) and SAHBNET (SA_c_ = 30%, R_c_ = 0.8 nm and K_SPRING_1000 kJ/nm^2^) for the simulations of the RNA-dependent RNA polymerases from human rhinovirus 16.

In their study, Shen *et al.* developed an ENM/MARTINI CG model and explored the dynamics of the RNA-dependent RNA polymerases. These proteins present multiple flexible regions previously described [36]. For the RNA-dependent RNA polymerase from human rhinovirus 16, the following stretches are expected to be flexible: 11–33, 48–66, 113–137, 153–178, 211–213, 257–261, 314–317, 381–387, 403–412, and 436–449. By using this information, we have evaluated SAHBNET on this protein. The RMSF of BB of the ELNEDYN and SAHBNET/MARTINI simulations are compared to the RMSF of the Cα of an atomistic simulation. The results are presented in Figure 5. All flexible parts of the protein are showing some flexibility in our model and their RMSF_Cα_ are close to the atomistic RMSF_Cα_, except for some residues in the zone 153–178, which are less flexible in our model. Compared to ELNEDYN, SAHBNET is closer to the flexibility of the atomistic structure and the local behaviors are mostly conserved (for example, residues 75–150) in the SAHBNET model. However, large movements found in the atomistic simulations (for example, residues around 175, or around 280) are not seen in the SAHBNET model, mainly due to the secondary structure constraints of the MARTINI model, which hinder secondary structure modifications.

### 2.4. Application of SAHBNET to Insert Membrane Proteins

#### 2.4.1. Insertion of PBP1b, a Monotopic Membrane Protein from *Escherichia coli*

As demonstrated in the previous sections, SAHBNET helps to keep the protein tertiary structure together with domain flexibility close to the atomistic one. Using SAHBNET with the parameters described above, PBP1b has been inserted in a membrane mimicking the natural one, containing 1,2-dioleoyl-sn-glycero-3-phosphoglycerol (DOPG), 1,2-dioleoyl-sn-glycero-3-phosphoethanolamine (DOPE), and cardiolipin. The preparation steps for that kind of protein are quite difficult and, to avoid some problems, we have used a modified version of genbox. The modifications made to the GROMACS tool and some of its applications are explained in Supplementary data S3.

**Figure 6:**
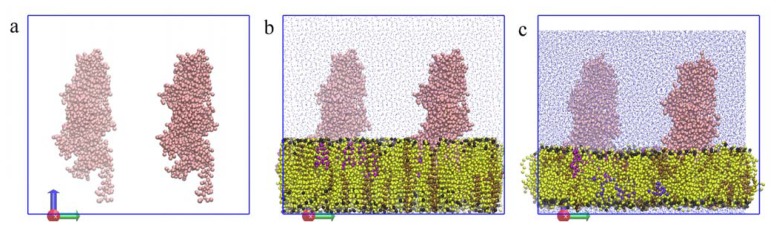
Illustration the different steps of the system preparation as described in Methods section. Phosphate group of the lipids are represented in black, 1,2-dioleoyl-sn-glycero-3-phosphoethanolamine (DOPE) molecules in yellow, 1,2-dioleoyl-sn-glycero-3-phosphoglycerol (DOPG) in brown, toy lipids in purple, water in blue and proteins in pink. (**a**) The proteins are inserted in the box; (**b**) The preorganized lipids were added in the correct space before solvation; (**c**) The system after 2.5 ns of simulation.

**Figure 7:**
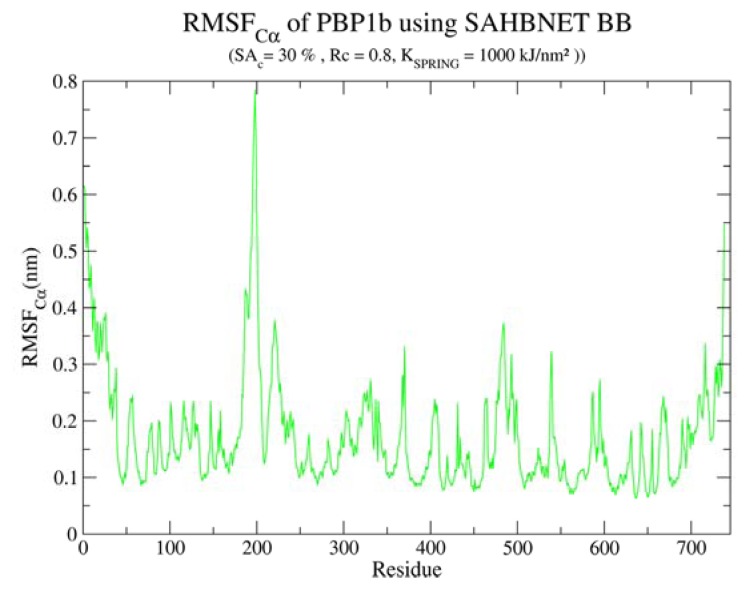
RMSF of the Cα of the monotopic membrane protein PBP1b using SAHBNET BB (SA_c_ = 30%, R_c_ = 0.8 nm and K_SPRING_1000 kJ/nm^2^).

Figure 6 illustrates the different steps of the system preparation. The protein is first positioned in a box, and the lipids are then positioned as described in methods. The protein structure is conserved and stable (data not shown). The flexibility of the protein was not compared against atomistic simulations but we have observed in the RMSF of the Cα (Figure 7), two highly flexible zones. The first one, between residues 1 and 40, corresponds to the transmembrane segment, and the second one, between residues 175 and 210, corresponds to an amphipathic loop. Both were expected to rearrange upon membrane interaction. Moreover, the amphipathic loop is expected to show some flexibility according to the catalytic model proposed for the protein [39].

#### 2.4.2. Application to the Outer Membrane Lipoprotein WZA

**Figure 8:**
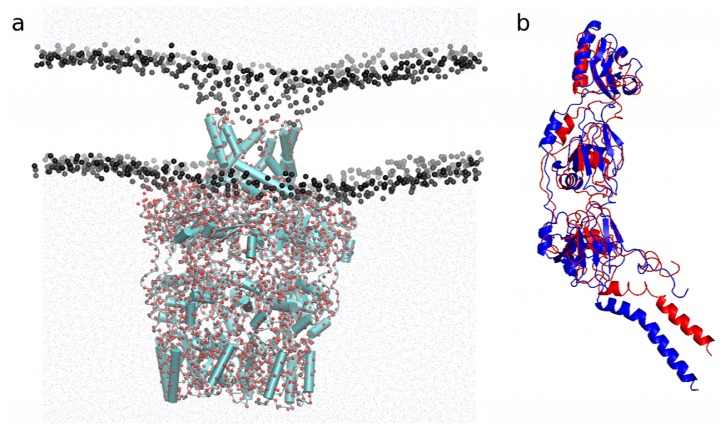
(**a**) Atomistic representation of the octamer of WZA inserted in the membrane. Phosphate group of the lipids are represented in black, secondary structures are represented in blue and the coarse-grained beads representing the final structure of the CG insertion dynamics are overplayed in red. The water is in light blue and the lipids are not shown excepted for the phosphate beads; (**b**) Comparison of one monomer before (blue) and after insertion (red).

We have also tested our method on the outer membrane lipoprotein WZA (PDB id: 2J58) in a DOPE:70/DOPG:30 membrane and run a 400 ns* simulation. The RMSD calculated from the initial structure for each WZA monomer remains stable and below ~4 Å along the simulation (data not shown); the global assembly is also stable. The protein is correctly inserted in the membrane and the lipids are perfectly equilibrated into a membrane around the hydrophobic core of the protein (Figure 8a). The hydrophobic mismatch at the interface between protein and lipids, and the resulting deformation of the membrane close to the periplasmic side of the protein, is due to the eight arginines pointing to the water. The structure of the octamer and one from a monomer after coarse-grained insertion procedure, and return to the atomistic representation using the method described by Rzepiele *et al.* [40] is shown in Figure 8b. The monomer was slightly deformed by the membrane insertion but the main secondary structures are still in place, especially the β-sheets which are difficult to maintain in the MARTINI representation.

## 3. Method

### 3.1. Molecular Systems

The villin headpiece subdomain (PDB [41] entry 1YRF [42]), the D48G mutant of the α-spectrin SH3 domain (PDB entry 1BK2 [43]), and the B1 domain of protein G (PDB entry 1PGB [44]) have been used in Periole *et al.* 2009 [35] to evaluate the ELNEDYN model and are used here to compare the SAHBNET with the ELNEDYN and atomistic models. The other proteins used in this study are: the RNA-dependent RNA polymerases from the human rhinovirus 16 (PDB entry: 1XR7); the penicillin binding protein PBP1b (PDB entry 3FWM [39]), a monotopic membrane protein from the bacterial divisome of *E. coli*; the outer membrane lipoprotein WZA (PDB entry: 2J58). Several lipid types have been used during this study. DOPC (1,2-dioleoyl-sn-glycero-3-phosphocholine), DPPE (1,2-dipalmitoyl-sn-glycero-3-phosphoethanolamine), and DMPC (1,2-dimyristoyl-sn-glycero-3-phosphocholine) have been used to build asymmetric membranes and vesicles, illustrating how the modified version of genbox works. DOPE (1,2-Dioleoyl-sn-Glycero-3-Phosphoethanolamine), DOPG (1,2-dioleoyl-sn- glycero-3-phosphoglycerol) and cardiolipin have been used to build a complex model membrane in which two PBP1b proteins are inserted. All lipids have been taken from the MARTINI’s website or made according to the topologies found in the forcefield.

### 3.2. Coarse-Grained Simulations

The MARTINI 2.1 forcefield was used for protein, lipid, ion and solvent description of the system and the GROMACS 4.5.4 program package was used to perform the molecular dynamic simulations. The CG proteins structure and topology were created using the latest MARTINI’s tools. When a protein is simulated without lipids, it is centered in a rectangular box with a minimum distance from the box edges of 1.2 nm. When a membrane has to be built, the proteins were centered inside a box and our modified version of genbox (described in the supplementary data) was used to randomly insert lipids close to the hydrophobic core of the protein. The number of lipids is computed from the XY surface of the box assuming that the surface of 256 lipids is approximately 100 nm^2^. After solvation, the systems were neutralized when needed by adding counter ions. Once the initial configuration has been built, all the systems studied were first energy-minimized, then a simulation of 1 ns with a time-step of 20 fs, and with position restraints on the backbone beads, was carried on. For ELNEDYN, a simulation of 50 ps with a time-step of 1 fs and with position restraints on all the protein beads was carried out. Finally, simulations without restraints were performed for 100 ns* or 400 ns* for longer time scales. As previously stated, a time correction has to be made in CG simulations with MARTINI forcefield, and all times corresponding to CG simulation denoted by a * are real time (4× simulation time). The lipids are allowed to self assemble while the protein structure is maintained by a position restraint.

As the tertiary protein structure is not stable in a MARTINI representation, the SAHBNET or ELNEDYN network was used to maintain protein structures during simulations. The SAHBNET intends to keep the hydrogen-bond network found in the atomistic structure and to insert springs between buried residues to complete the MARTINI secondary structure network. The SAHBNET is used during the minimization, position restraint, and production run. Hydrogen-bonds and solvent accessible surface area (SA) per residue are taken from the output of Stride [45]. The SA was normalized against the mean SA obtained from stride for the central residue of the 400 petapetides of the following sequence GX_1_RX_2_G (X = any amino-acid, R = the measured residue) in extended form. The mean SA is given in Table 1.

Two backbone beads are linked by a spring with a force constant, K_SPRING_, if their atomistic counterparts are involved in a hydrogen-bond. Backbone (BB) beads or side-chains (SC) beads from a residue with an accessible surface smaller than a defined cut-off (SA_c_) are linked by a spring with a force constant, K_SPRING_, to BB, or SC beads only if the distance between them in the experimental structure is less than a predefined cut-off, R_c_, and if they are at least separated by five positions in the protein sequence. During the CG simulations, the temperature and pressure were maintained at 300 K and 1 bar by using the bath method of Berendsen [46] with time constants of 0.5 ps (τ_T_) and 1.2 ps (τ_P_). The non bonded interactions were treated with a switch function from 0.9 to 1.2 nm for Lennard-Jones interactions and one from 0.0 to 1.2 nm for Coulomb interactions. The integration time-step was set to 20 fs and the neighbor list was updated every five steps. As already described, the time scales are faster in CG simulations due to the smoothing of the energy surface and a correction factor of four for the time is then used.

A script has been written to automatize systems preparation based on the MARTINI forcefield and is usable through the E-GCGS website (gcgs.gembloux.ulg.ac.be). This website allows the use of the modified version of genbox and SAHBNET.

### 3.3. Comparison between SAHBNET, ELNEDYN and Atomistic Simulations

To study the effect of the three variables SA_c_, R_c_, and K_SPRING_ on the flexibility and structural properties of the proteins with SAHBNET, the value of these variables were varied from 10% to 50%, 0.4 to 1.0 Å, and 100 to 10,000 kJ mol^−1^ nm^−2^, respectively. The effects of the network on the protein structure were compared by using the RMSD and RMSF computed on the Cα, as well as delta maps computed between the mean RMSD or RMSF of the coarse-grained and atomistic simulations. For the atomistic simulations, the last 60 ns were considered, while for the coarse grained simulations it was the last 15 ns (60 ns in real time). The number and location of springs were also compared.

### 3.4. Atomistic Simulations

Simulations have been performed with the Gromos96 43a1 forcefield [47] and the SPC water model is used for the solvation. The proteins were placed in a rectangular box with a minimum distance from the box edges of 1.0 nm. After solvation, the systems were neutralized when needed by adding counter ions. All the systems studied were first energy-minimized then two simulations of 10 ps with position restraints were carried out. The first one with the position restraints on the heavy atoms of the protein and the second one on the Cα. Runs without restraints were performed for 100 ns. The temperature and pressure were maintained at 300 K and 1 bar by using the bath method of Berendsen [46] with time constants of 0.1 ps (τ_T_) and 1.0 ps (τ_P_). The integration time-step was set to 2 fs. Non-bonded interactions were treated by using a twin range cutoff (1.0–1.4 nm) with the interactions which are within the long-range cutoff evaluated every five time steps. A Reaction-Field [48] correction (ɛ = 78) was applied for the electrostatic interactions beyond this cutoff. The protein and water bond lengths were maintained with the LINCS [49] and SETTLE [50] algorithms respectively. The simulations were performed and analyzed with the GROMACS 4.5.5 tools as well as with homemade scripts and software. The last 60 ns were used in the analyses, and 3D structures were analyzed with PYMOL and VMD software [51,52].

### 3.5. Modifications of Genbox

Genbox is a tool of GROMACS that allows the filling of empty space in a box with solvent molecules, which can be water or lipids, for example. A small set of modifications has been made in the source code of genbox to easily set up complex membrane compositions and deal with periodic boundary condition artifacts. The *iBox*, *tBox* and *norot* options have been added and stand for “insertion box”, “translation box”, and “no rotation” respectively. *iBox* is a box of a given size centered in the initial box, in which the lipids can be inserted. *tBox* allows the translation of the *iBox* in the three dimensions. *norot* removes the rotation of the molecule inserted in the box around *x* and *y* axes. Since a *norot* like option will be found in the future versions of GROMACS (and is called -rot), the patch only includes the *iBox* and *tBox* options, and can be found on our website (gcgs.gembloux.ulg.ac.be/downloads). It should be mentioned that for larger systems, a workaround for the memory leak exists in GROMACS developers’ version.

## 4. Conclusions

The SAHBNET method allows significant conservation of the structure of proteins or protein domains with only few springs. We have calibrated SAHBNET on different soluble proteins and demonstrated that it can be helpful to maintain protein structure and flexibility. It uses information from the hydrogen-bond network and the residue accessible surface area to improve the formation of an elastic network and to maintain the coarse-grained structure. It appears to be a good alternative to other elastic networks like ELNEDYN. Moreover, using SAHBNET we have demonstrated that the number of bonds required for maintaining the structure in elastic network models is probably overestimated. Finally, we have shown that SAHBNET can be applied to complex membrane protein simulations, as demonstrated respectively with PBP1b and the WZA octamer.

## Supplementary Information



## Figures and Tables

**Table 1:** Mean solvent accessible surface area per residue used for normalization.

Residue	Mean solvent accessible surface area
A	103.8
R	231.1
N	157.6
D	156.7
C	130.8
E	195.0
Q	195.7
G	80.8
H	180.4
I	168.5
L	171.5
K	206.4
M	190.6
F	198.2
P	121.6
S	123.5
T	138.6
W	229.6
Y	219.7
V	144.7
