# Supplementary material for: SAHBNET, an Accessible Surface-Based Elastic Network: An Application to Membrane Protein
Source: Int J Mol Sci. 2013 May 30;14(6):11510–26. doi: 10.3390/ijms140611510 (PMC3709745; doi:10.3390/ijms140611510)
Supplement: Supplementary file 1 [file ijms-14-11510-s001.pdf]

Supplementary Information

Table S1. Number of residues, hydrogen bonds and ELNEDIN springs for each protein.

Proteins (PDB entry)	Residues	H-bonds	ELNEDIN springs
B1 domain of protein G (1PGB [46])	56	42	202
Villin headpiece subdomain (1YRF [47])	35	25	100
D48G mutant of the α-spectrin SH3 domain(1BK2 [48])	57	28	229
Penicillin binding protein PBP1b (3FWM [49])	738	453	3428

Table S2. Number of springs generated to make the SAHBNET without the hydrogen-bonds related springs according to the protein, SAc and Rc. NC: Not Calculated, BB: Backbone Beads, SC: Side-Chains.

		Number of SA based springs							
		SAHBNET BB				SAHBNET SC			
SA_c(%)	R_c (nm)	1PGB	1YRF	1BK2	3FWM	1PGB	1YRF	1BK2	3FWM
10	0.4	NC	NC	NC	21	0	1	2	19
	0.5	5	0	9	72	10	13	19	215
	0.6	10	0	22	232	31	21	42	519
	0.7	19	2	34	444	NC	NC	NC	812
	0.8	28	4	43	719	NC	NC	NC	1144
	0.9	42	5	58	1174	NC	NC	NC	1640
	1	64	5	82	1963	NC	NC	NC	2390
20	0.4	NC	NC	NC	25	0	2	2	32
	0.5	5	0	10	96	10	18	21	302
	0.6	10	0	25	301	31	37	48	705
	0.7	20	2	39	576	NC	NC	NC	1109
	0.8	29	4	49	930	NC	NC	NC	1582
	0.9	43	8	65	1541	NC	NC	NC	2279
	1	66	13	94	2552	NC	NC	NC	3339
30	0.4	NC	NC	NC	28	1	2	2	39
	0.5	8	0	13	105	13	18	30	345
	0.6	16	1	35	330	43	37	69	800
	0.7	29	3	61	649	NC	NC	NC	1270
	0.8	41	6	78	1061	NC	NC	NC	1826
	0.9	64	11	101	1782	NC	NC	NC	2633
	1	105	17	146	2951	NC	NC	NC	3858

S3. Modifications Made to the Genbox Gromacs Tool

In the "self-assembly" method described by Bond *et al.* in 2006 [1], the first steps of the system preparation consist in the positioning of the peptide or protein in a box which is then filled with lipids. After an increase of the box Z axis, the new empty space is filled with water molecules. The insertion of lipid and water particles can be done by using genbox found in Gromacs's tools set. With genbox, each molecule that has to be inserted is randomly rotated around the *X*, *Y* and *Z* axes and positioned at a random position in the box. If there is not enough space, the program tries another set of coordinates. These steps are repeated until a place has been found for each molecule. If this tool is suitable to prepare simple membranes and to simulate the insertion of peptides or small proteins in a membrane, it often fails to form a membrane in presence of a monotopic membrane protein. If the initial box that will be filled with lipids has the size of the protein, a membrane will never form at the level of the transmembrane segment because lipids are too scattered in the box. If the intial box is centered at the level of the transmembrane segment, there will be a lipid void around the transmembrane segment due to the periodic boundary conditions (pbc). When the protein is bigger than the initial box, it will fill the whole space where lipids would normally be inserted. The membrane will take a longer time to equilibrate and in some cases box collapses or abnormal assembly of lipids can occur (Figure S1). To avoid those problems, we have added new options to genbox: *iBox* and *tBox*, respectively insertion box and translation box and *norot* for removing the rotation of the molecule around *X* and *Y* axes. *iBox* is a box of a given size centered in the initial box in which the lipids will be inserted. *tBox* allows the translation of the *iBox* in the three dimensions. A patch for genbox with the *iBox* and *tBox* options can be found on our website (gcgs.gembloux.ulg.ac.be/downloads). Using this modified version of genbox, any protein whatever its size can be placed in the final sized box, lipids can be added where needed and water anywhere else. The modified version of genbox can be used to make different lipid assemblies (Figure S2). For example, by using the *iBox* option, we can localize the lipids in the middle of the box and in the same time inserted water around the lipids. After a few nanoseconds of dynamics, a membrane has been formed. Lipids from the top and bottom leaflets can be inserted separately. The *iBox*, *tBox* and *norot* options were then used to insert lipids aligned parallel and anti-parallel to the Z axis respectively for top and bottom leaflet. Using this two-steps method, we easily create asymmetric membranes (Figure S2a). Without the *norot* option, multiple vesicles can also be obtained (Figure S2b).

Each time, the membrane rapidly equilibrates (usually 2.5 ns) with less than 1% deviation from the desired composition for each leaflet. With these new options of genbox, it's possible to prepare easily almost any protein-lipid system.

Figure S1. Illustration of an abnormal lipid assembly around the monotopic protein PBP1b. The protein is colored in orange except for the transmembrane segment in red. Lipids are in blue except for the phosphate group in black. (**a**) Before equilibration; (**b**) After 10 ns simulation, the box has collapsed on the *X* and *Y* axes and a hole is still present in the membrane.

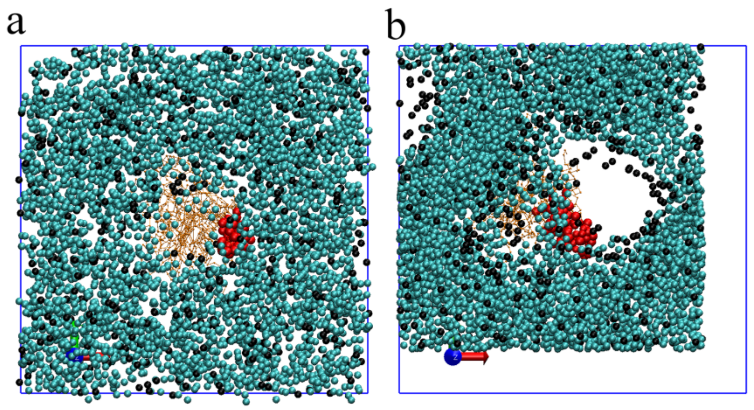

Figure S2. Illustration of the use of the modified genbox version for the preparation of an asymmetric membrane with 128 DOPC (red) and 128 DPPE (green). The membrane is solvated with 4158 CG Water (blue). (**a**) Before and (**b**) after 2.5 ns equilibration. Vesicles with each 256 DMPC (orange) and 51 DPPE before (**c**) and after (**d**) 2.5 ns of simulation. The vesicles are solvated with 53312 CG water. Beads representing the phosphate group of the lipids are colored in black.

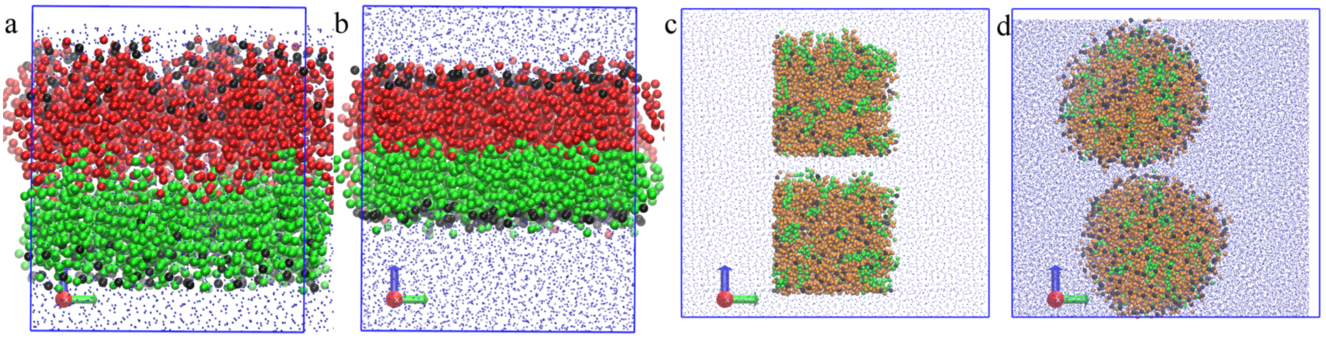

Reference

1. Bond, P.J.; Sansom, M.S.P. Insertion and assembly of membrane proteins via simulation. *J. Am. Chem. Soc.* **2006**, *128*, 2697–704.

© 2013 by the authors; licensee MDPI, Basel, Switzerland. This article is an open access article distributed under the terms and conditions of the Creative Commons Attribution license (http://creativecommons.org/licenses/by/3.0/).
